# Acknowledgement to Reviewers of *Vision* in 2019

**DOI:** 10.3390/vision4010008

**Published:** 2020-01-19

**Authors:** Vision Editorial Office

**Affiliations:** MDPI, St. Alban-Anlage 66, 4052 Basel, Switzerland

The editorial team greatly appreciates the reviewers who have dedicated their considerable time and expertise to the journal’s rigorous editorial process over the past 12 months, regardless of whether the papers are finally published or not. In 2019, a total of 71 papers were published in the journal, with a median time to first decision of 33.05 days and a median time from submission to publication of 80 days. The editors would like to express their sincere gratitude to the following reviewers for their generous contribution in 2019:
Al-Janabi, ShahdKuperman, VictorAllison, RobertLages, MartinAlzahabi, ReemLappin, Joseph S.Ansorge, UlrichLatimer, Cyril R.Anstis, StuartLea, BrookeAsher, Jordi M.Legras, RichardBaker, Curtis L.Levitt, Jonathan B.Baker, Daniel H.Li, YunghuiBarrett, DougLisi, MatteoBattaglini, LucaLitchfield, DamienBayless, SarahLiu, QuanBelardinelli, AnnaLunghi, ClaudiaBengson, Jesse J.Lupi, LiviaBerlucchi, GiovanniMacKenzie, AndrewBernhardt-Walther, DirkMadokoro, HirokazuBlythe, Hazel I.Markovic, SlobodanBorn, SabineMarkowitz, SamuelBrascamp, Jan W.Mather, GeorgeBreitmeyer, BrunoMathôt, SebastiaanBrenner, EliMaule, JohnBurkhalter, AndreasMedley, StuartCampana, GianlucaMeduri, AlessandroCamponogara, IvanMeier, KimberlyCastelhano, Monica S.Meso, AndrewCave, KyleMilledge, SaraChakraborty, ArijitMinissale, GregoryChakraborty, ArijitMirpour, KooroshChukoskie, LeanneMuhl-Richardson, AlexClarke, AlasdairMulckhuyse, ManonClement, JesperNah, Joseph C.Cole, GeoffNava, ElenaColenbrander, AugustNiehorster, Diederick C.Coppola, GianlucaNiemeier, MatthiasCoulson, SeanaNoel, Jean PaulCumming, BruceOgmen, HalukDaini, RobertaO’Hare, LouiseDe Graaf, Tom AlexanderPapenmeier, FrankDe Haan, Alyanne M.Parks, Theodore E.De Winkel, KsanderPaterson, KevinDieni, CristinaPeirce, JonDijkstra, NadinePinet, SvetlanaDimigen, OlafPoletti, MartinaDonnelly, NickPopelka, StanislavDoyle, LesleyPoth, ChristianDrieghe, DenisPrins, NicolaasDumont, JulieRafal, RobertEdwards, GarethRead, JennyEinhäuser-Treyer, WolfgangReeves, AdamEl Shamieh, SaidRemington, RogerEnns, JamesRezazadegan Tavakoli, HamedFarell, BartRichard, BrunoFeldmann-Wüstefeld, TobiasRizvi, Syed Tahir HussainFiehler, KatjaSagi, DovFoulsham, TomSahraie, ArashFranceschini, SandroSánchez-Ávila, Ronald MauricioFrank, SebastianSandin, FredrikFrench, RobertSapir, AyeletFriedenberg, JaySawada, TadamasaGabriel, MaximilianSchallmo, Michael-PaulGarweg, Justus G.Schütz, AlexanderGeorgeson, MarkSchwiedrzik, Caspar M.Gibaldi, AgostinoSheridan, HeatherGinis, HarilaosSiegert, IngoGodwin, Hayward J.Sigurdardottir, Heida MariaGoffart, LaurentSimpson, BrianGoodale, MelvynSmith, DanGrisanti, SalvatoreSoranzo, AlessandroGu, YongSorrentino, Francesco SaverioHahn, BrittaSpehar, BrankaHamm, Jeff P.Spiller, MaryHarezlak, KatarzynaStevens, RebekahHarvey, BenStringham, JamesHashemi, AliSun, YankuiHauk, OlafTagu, JérômeHavig, PaulTakao, MotoharuHayward, Dana A.Tiffin-Richards, SimonHeller, JanoschTünnermann, JanHessels, Roy S.Van Den Berg, TJTPHeuer, AnnaVan Der Stigchel, StefanHibbard, PaulVasilev, MartinHipsley, AnnMarieVeldre, AaronHolmes, NicholasVerghese, PreetiHoupt, JoeVo, MelissaHout, MichaelWade, NicholasHübner, RonaldWalenchok, StephenHulleman, JohanWalker, RobinInhoff, AlbrechtWard, JamieJabar, SyaheedWarrington, KaylieghJayakumar, ManasiWaugh, SarahJiang, FangWhite, SarahJohnson, JeffWilber, Aaron A.Joshi, Mahesh RajWilliams, CarrickKello, ChrisWolffsohn, James J.Kerzel, DirkYadav, SrishtiKiyonaga, AnastasiaZang, ChuanliKlin, CeliaZhang, TaoKuhn, GustavZhang, KangKulke, Louisa


